# The effectiveness of physiotherapy interventions on fecal incontinence and quality of life following colorectal surgery: a systematic review and meta-analysis of randomized controlled trials

**DOI:** 10.1007/s00520-023-08294-1

**Published:** 2024-01-13

**Authors:** Ming Yan Pun, Pak Ho Leung, Tsz Ching Chan, Chunn Pang, Kin Hei Chan, Priya Kannan

**Affiliations:** Department of Rehabilitation Sciences, The Hong Kong Polytechnic University, Hung Hom, Kowloon, Hong Kong

**Keywords:** Biofeedback, Colorectal cancer, Colorectal surgery, Fecal incontinence, Pelvic floor muscle training, Physiotherapy, Quality of life

## Abstract

**Purpose:**

To investigate the effectiveness of physiotherapy interventions compared to control conditions on fecal incontinence (FI) and quality of life (QoL) following colorectal surgery.

**Methods:**

Electronic searches in English-language (Scopus, Web of Science, Embase, AMED, CENTRAL, CINAHL, MEDLINE, Ovid, and PEDro) and Chinese-language (CNKI, Wanfang Data) databases were conducted. Trials comparing physiotherapy interventions against control conditions and assessing FI and QoL outcomes were included in the review.

**Results:**

Ten trials were included. Meta-analysis revealed statistically significant improvements in lifestyle (0.54; 95% CI 0.03, 1.05; *p* = 0.04), coping behavior (MD 1.136; 95% CI 0.24, 2.04; *p* = 0.01), and embarrassment (0.417; 95% CI 0.14, 0.70; *p* = 0.00) components of QoL among individuals receiving pelvic floor muscle training (PFMT) compared with those receiving usual care (UC). Meta-analysis showed biofeedback to be significantly more effective than UC in enhancing anal resting pressure (ARP; 9.551; 95% CI 2.60, 16.51; *p* = 0.007), maximum squeeze pressure (MSP; 25.29; 95% CI 4.08, 48.50; *p* = 0.02), and rectal resting pressure (RRP; 0.51; 95% CI 0.10, 0.9; *p* = 0.02). Meta-analysis also found PFMT combined with biofeedback to be significantly more effective than PFMT alone for ARP (3.00; 95% CI 0.40, 5.60; *p* = 0.02), MSP (9.35, 95% CI 0.17, 18.53; *p* = 0.05), and RRP (1.54; 95% CI 0.60, 2.47; *p* = 0.00).

**Conclusions:**

PFMT combined with biofeedback was more effective than PFMT alone, but both interventions delivered alone were superior to UC. Future studies remain necessary to optimize and standardize the PFMT parameters for improving QoL among individuals who experience FI following CRC surgery.

**Review registration:**

This systematic review is registered in the PROSPERO registry (Ref: CRD42022337084).

**Supplementary Information:**

The online version contains supplementary material available at 10.1007/s00520-023-08294-1.

## Background

Colorectal cancer (CRC) is more common among men than women, and as of 2020, CRC had a 5-year prevalence rate of 51.9 per 100,000 people in Asia and an incidence rate of 68 per 100,000 in Hong Kong [1, 2]. Surgical methods are commonly applied to remove tumors from the colon or rectum, including low anterior resection (LAR), laparoscopy, abdominoperineal resection, and transanal microsurgery. However, fecal incontinence (FI), defined as the “involuntary loss of feces when feces are solid and/or liquid,” commonly develops following surgical management of CRC and chemoradiation [3]. Following surgery, FI can develop due to damage to muscular, fascial, or neural tissues during surgery [4]. Seventy to 90% of the patients who undergo sphincter preserving surgery experience FI in addition to other symptoms such as incontinence for flatus, increased intestinal gas, and rectal urgency (commonly referred to known as LAR syndrome) [5]. The reported incidence of FI in patients treated with pelvic chemoradiation ranges between 3 and 53% [6, 7]. FI has a significant negative impact on the quality of life (QoL) among CRC survivors [8]. The inability to control the involuntary leakage of stool can cause embarrassment and fear of such episodes may hinder social participation or physical activity, adversely affecting mental health [9]. Therefore, the post-surgical management of FI is necessary.

Treatments for FI include both pharmaceutical and non-pharmaceutical interventions. Non-pharmaceutical treatments may include diet adjustments, anal plugs, or physiotherapy [10, 11]. Physiotherapy interventions for the treatment of FI include pelvic floor muscle training (PFMT) with or without biofeedback, neuromuscular electrical stimulation (NMES), and acupuncture [12]. A recent review by Kim and Oh [13] evaluated the effectiveness of PFMT on bowel function and health-related QoL among patients who have undergone LAR and included studies published until 2019. Since then, further studies have been published on physiotherapy interventions in the management of FI, and therefore, the review requires updating. The objective of this review is to investigate the effectiveness of physiotherapy interventions on FI and the QoL following colorectal surgery.

## Methods

This systematic review and meta-analysis was conducted according to the Preferred Reporting Items for Systematic Reviews and Meta-Analyses (PRISMA) guidelines [14] and was registered in the International Prospective Register of Systematic Reviews (PROSPERO, CRD42022337084).

### Search strategy

Electronic databases of English-language articles, including the Allied and Complementary Medicine Database (AMED), The Cochrane Central Register of Controlled Trials (CENTRAL), the Cumulative Index of Nursing and Allied Health (CINAHL), Embase, MEDLINE, Ovid, the Physiotherapy Evidence Database (PEDro), Scopus, and Web of Science, and electronic databases of Chinese-language articles, including Chinese National Knowledge Infrastructure (CNKI) and Wanfang Data, were searched to identify potentially relevant articles. Searches were conducted from database inception to December 2021 and updated in November 2022. Searches were conducted by two independent review authors (P.C. and C.T.C). The specific search strategy applied to the Medline database is presented in Supplementary Appendix 1. Four themes, “physiotherapy intervention,” “colorectal cancer and surgery,” “bowel incontinence,” and “randomized controlled trials,” were utilized to identify potentially relevant articles. Related terms associated with each theme were combined using the Boolean operator “OR.” All four themes were combined using the Boolean operator “AND.” EndNote 20 citation management software was used to archive and organize the search results and remove duplicates. Manual searches were also performed by examining the reference lists for each included trial and relevant systematic reviews to identify additional candidate trials.

### Study eligibility criteria

Trials were included if they (1) were randomized controlled trials (RCTs, including pilot, cluster, or crossover trials) comparing physiotherapy interventions (acupuncture, biofeedback, electrical stimulation, aerobic exercises, resistance exercises, stretching exercises, manual therapy, PFMT, or yoga) with a control condition (no treatment, usual care [UC], placebo, or active control) on FI and QoL outcomes following colorectal surgery; (2) measured FI using the Cleveland Clinic Incontinence Score (CCIS, also known as the Wexner score), the low anterior resection syndrome (LARS) score, or anorectal manometry (ARM) or measured QoL using the Fecal Incontinence Quality of Life (FIQL) scale, the EuroQoL 5-Dimension (EQ-5D) scale, the European Organization for Research and Treatment of Cancer (EORTC), Quality of Life Questionnaire (QLQ) module for colorectal cancer (QLQ-CR29), the EORTC QLQ module for cancer (QLQ-C30), or the 36-Item Short Form Survey (SF-36); and (3) were available in full-text format in either English or Chinese (traditional or simplified). Unpublished theses were also included in the review if they met the review criteria described above. Studies were excluded if they were systematic reviews, RCT/systematic review protocols, or case reports. Trials that included subjects with FI associated with causes other than colorectal surgery or radiotherapy for CRC (such as labor, prostate cancer, malabsorption, spinal surgery, or congenital disorders) were excluded. Trials reporting data as median and interquartile range, trials that included subjects with FI secondary to other medical conditions, such as neurological diseases, and trials that evaluated the effectiveness of non-physiotherapy treatments (such as anal plugs, Chinese medication, herb medication, injection, acupressure, moxibustion) were also excluded.

### Article screening

Articles identified through the electronic searches underwent a three-stage screening process, including title, abstract, and full-text screening. Studies were screened for inclusion by two reviewers (P.M.Y. and L.P.H.). Disagreements between reviewers were resolved by discussion, and a third reviewer (P.K.) was consulted if the disagreement remained unresolved after discussion.

### Data extraction

Data extraction was conducted independently by three authors (P.C., P.K., and C.K.H). The following data were extracted from each included study: last name of the first author, publication year, country of origin, sample size, mean age/age range of study subjects, intervention and control, outcome measures, and pre- and post-treatment data (mean and standard deviation) of ARM measured by anal resting pressure (ARP) and maximum squeeze pressure (MSP) for FI, and lifestyle, coping behavior, depression, and embarrassment components of QoL. For post-treatment data, only the data associated with the longest follow-up period was extracted.

### Quality assessment

Five authors (P.C., P.K., C.K.H., P.M.Y., and L.P.H) independently assessed the methodological quality of the included trials using the Revised Cochrane Risk-of-Bias (RoB 2) tool for randomized trials [15]. Disagreements between reviewers were resolved by discussion. An additional reviewer (PK) was consulted for any unresolved disagreements. The RoB 2 categorizes the overall RoB level as high, moderate, or low, based on the RoB identified in five domains: (1) randomization process, (2) deviation from intended interventions, (3) missing outcome data, (4) outcome measurements, and (5) the selection of the reported results [15]. The overall RoB evaluation was guided by signaling questions in the five domains [15].

### Data synthesis and statistical analysis

All meta-analyses were performed using Comprehensive Meta-Analysis software (CMA version 3.3.070, Biostat Inc., Englewood, NJ, USA). Statistical heterogeneity was measured using the *I*^2^ test. A random-effect model was applied for high heterogeneity (*I*^2^ > 50%); otherwise, a fixed-effect model was utilized [16]. Trials evaluating similar physiotherapy interventions, control conditions, and outcome measures were grouped together for meta-analysis. For continuous data, the weighted mean difference (WMD) and 95% confidence interval (CI) were calculated. WMD was chosen because similar units of measurement were used for outcome measures in the included trials [17]. In the meta-analyses and throughout the “Results” section, all data for ARP and MSP reported by Chen [18] were converted from mmHg to kPa (by multiplying by 0.133) [19] to standardize the unit for the calculation of WMD. Significance was defined as a *p*-value ≤ 0.05.

## Results

**Fig. 1:**
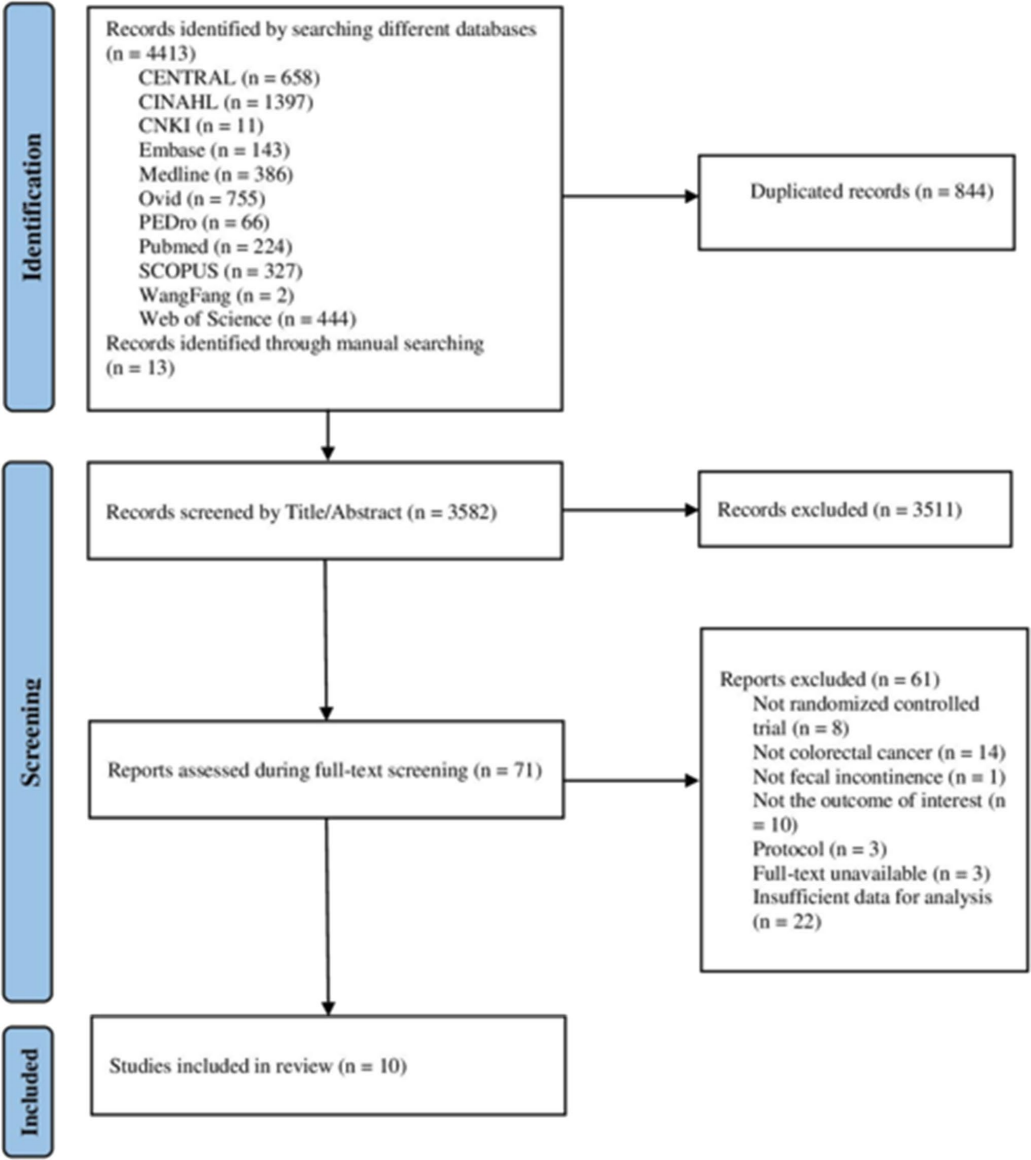
Flow diagram of the study selection process applied for this review

The study selection process, which followed the PRISMA approach, is summarized in Fig. 1. The studies that were excluded at the full-text screening stage and the reasons for exclusion are listed in Supplementary Appendix 2. A total of 4413 articles were identified via electronic and manual searches. After all screening stages were applied, only 10 trials met the inclusion criteria for the meta-analytic review.

### Characteristics of the included trials

Table 1 summarizes the characteristics of the included trials. Across all 10 trials, data were extracted for 608 subjects. The sample sizes of the included trials ranged from 12 to 100. The mean age of participants in the included trials ranged from 45.6 to 66.8 years. All subjects in the included studies had fecal incontinence following surgery for CRC. The interventions assessed in the included trials were PFMT (*n* = 2), biofeedback (*n* = 4), and biofeedback combined with PFMT (*n* = 4). Among the 10 included trials, three trials reported QoL outcomes, and eight trials reported FI outcomes.

**Table 1:** Characteristics of included trials (*n* = 10)

First author, year, country of study	Mean age of participants (SD); sample size of each group	Intervention	Control	Outcome measure(s)	Results (time points of assessment): mean (SD)
Chai [23], 2018, China	Exp: 62.9 (8.6) Con: 62.7 (6.7) Exp: *n* = 46 Con: *n* = 28	BFT - 3 months Details NR	Usual care	FI: ARM (ARP, MSP)	*ARM: ARP (mmHg)* Pre Exp: 56.4 (8.7) Con: 56.4 (8.7) Post (12 months) Exp: 47.6 (8.0) Con: 28.7 (7.9) *ARM: MSP (mmHg)* Pre Exp: 234.2 (31.7) Con: 244.2 (28.7) Post (12 months) Exp: 189.9 (36.4) Con: 131.3 (36.1)
Chen [18], 2021, China	Exp: 56.28 (8.07) Con: 56.22 (8.14) Exp: *n* = 38 Con: *n* = 38	PFMT - Contract pelvic floor muscle and hold for 5–10 s, rest for 10 s - 10 reps/set × 5 sets/day - Start on post-op 2nd week until discharge EMG-BFT - 20–30 min/session × 3 sessions/week Start on post-op 2nd week until discharge	PFMT - Contract pelvic floor muscle and hold for 5–10 s, rest for 10 s - 10 reps/set × 5 sets/day Start on post-op 2nd week until discharge	FI: ARM (ARP, MSP)	*ARM: ARP (mmHg)* Pre Exp: 25.88 (15.83) Con: 26.18 (15.75) Post (on discharge) Exp: 56.85 (18.45) Con: 51.15 (17.78) *ARM: MSP (mmHg)* Pre Exp: 71.86 (29.40) Con: 72.00 (29.33) Post (on discharge) Exp: 138.39 (33.53) Con: 115.28 (34.65)
Cho [28], 2021, Korea	Exp: 61.7 (9.8) Con: 64.5 (9.4) Exp: *n* = 21 Con: *n* = 26	BFT - 1–2 times/week × 6 months PFMT Details NR	PFMT Details NR	FI: CCIS	*CCIS* Pre NR Post (12 months) Exp: 10.05 (5.2) Control: 10.17 (5.3)
Ng [20], 2016, Taiwan	All participants: 66.8 (12.5)* Exp: *n* = 26 Con: *n* = 26	PFMT 20 reps/session × 4 sessions/day × 9 months	Usual care	QoL: FIQL	*FIQL: Lifestyle* Pre Exp: 3.29 (0.87) Con: 2.99 (0.98) Post (6 months) Exp: 3.68 (0.55) Con: 3.66 (0.68) *FIQL: Coping behavior* Pre Exp: 3.41 (0.81) Con: 2.85 (1.04) Post (6 months) Exp: 3.60 (0.68) Con: 3.72 (0.64) *FIQL: Depression* Pre Exp: 3.30 (0.50) Con: 3.19 (0.61) Post (6 months) Exp: 3.43 (0.40) Con: 3.44 (0.49) *FIQL: Embarrassment* Pre Exp: 3.61 (0.71) Con: 3.45 (0.71) Post (6 months) Exp: 3.73 (0.53) Con: 3.85 (0.38) *FIQL: Total* Pre Exp: 13.62 (2.50) Con: 12.50 (2.93) Post (6 months) Exp: 14.44 (1.85) Con: 12.50 (2.06)
Kim [27], 2015, Korea	Exp: 60.6 (6.0) Con: 54.5 (10.1) Exp: *n* = 6 Con: *n* = 6	BFT - Visual, auditory, and verbal biofeedback - Subjects were instructed to breathe naturally without stopping during the pelvic muscle contraction exercise and to slowly contract and hold the muscle tightly, followed by a break - Strength and the number of exercises gradually increased - Twice per week before surgery Lasted 4 weeks after surgery	PFMT Details NR	FI: CCIS QoL: FIQL	*CCIS* Pre Exp: 9.0 (3.5) Con: 10.7 (3.1) Post (12 months) Exp: 6.2 (3.1) Con:7.0 (3.5) *FIQL: Lifestyle* Pre Exp 22.2 (10.4) Con: 20.5 (11.0) Post (12 months) Exp: 32.8 (4.7) Con: 30.8 (8.1) *FIQL: Coping behavior* Pre Exp 20.0 (7.3) Con: 17.3 (8.0) Post (12 months) Exp: 26.5 (5.4) Con: 26 (8) *FIQL: depression* Pre Exp 17.8 (6.2) Con: 14.3 (5.4) Post (12 months) Exp: 16.7 (4.2) Con: 16.2 (3.2) *FIQL: embarrassment* Pre Exp 8.5 (3.2) Con: 8.3 (3.0) Post (12 months) Exp: 10.2 (1.7) Con: 10.0 (2.1)
Xia [21], 2016, China	Exp: 58.6 (11.4) Con: 60.5 (12.1) Exp: *n* = 30 Con: *n* = 30	PFMT - Hold > 10 s, rest > 10 s 20 reps/set × 3 sets/day × 12 weeks	Usual care	QoL: FIQL	*FIQL: Lifestyle* Pre Exp: 2.4 (0.7) Con: 2.6 (0.6) Post (3 months) Exp: 3.6 (1.1) Con: 3.0 (0.9) *FIQL: Coping behavior* Pre Exp: 2.8 (0.9) Con: 2.9 (1.0) Post (3 months) Exp: 3.9 (1.0) Con: 3.4 (0.9) *FIQL: Depression* Pre Exp: 2.6 (1.0) Con: 2.8 (0.8) Post (3 months) Exp: 3.9 (1.2) Con: 3.3 (1.0) *FIQL: Embarrassment* Pre Exp: 2.7 (0.8) Con: 2.8 (0.9) Post (3 months) Exp: 3.9 (1.1) Con: 3.3 (1.0)
Yang [25], 2020, China	Exp: 61.31 (10.05) Con: 61.26 (10.05) Exp: *n* = 32 Con: *n* = 32	EMG-BFT - 20 min/day × 3 days/week × 3 months PFMT - Contract pelvic floor muscles in a comfortable position for 10 s repeatedly until post-op 16 weeks on rest days between sessions	PFMT - Contract pelvic floor muscles in a comfortable position for 10 s repeatedly until post-op 16 weeks on rest days between sessions	FI: ARM (ARP, MSP)	*ARM: ARP (mmHg)* Pre Exp: 45.32 (8.95) Con: 45.26 (9.01) Post Exp: 44.21 (7.41) Con: 40.26 (8.15) *ARM: MSP (mmHg)* Pre Exp: 131.89 (11.03) Con: 132.56 (10.59) Post Exp: 129.64 (11.03) Con: 120.69 (10.53)
You [24], 2018, China	All subjects: 53.6 (3.7)* Exp: *n* = 26 Con: *n* = 26	BFT Post-op 1st month until post-op 6th month	No treatment	FI: ARM (ARP, MSP, RRP)	*ARM: ARP (mmHg)* Pre Exp: 31.26 (6.15) Con: 29.72 (5.61) Post (6 months) Exp: 42.07 (4.97) Con: 35.49 (7.13) *ARM: MSP (mmHg)* Pre Exp: 116.34 (16.97) Con: 117.42 (16.81) Post (6 months) Exp: 141.16 (5.28) Con: 133.24 (4.89) *ARM: RRP (mmHg)* Pre Exp: 7.56 (1.39) Con: 7.81 (1.93) Post (6 months) Exp: 6.07 (0.64) Con: 7.21 (0.87)
Zhang [22], 2016, China	All subjects: 45.6 (6.7)* Exp: *n* = 50 Con: *n* = 50	BFT - Adhesive electrodes on the external oblique abdominis and anal electrode for the external anal sphincter 45–60 min/day × 1 month	Usual care	FI: ARM (ARP, MSP, RRP)	*ARM: ARP (mmHg)* Pre Exp: 33.52 (3.45) Con: 32.67 (3.51) Post (9 months) Exp: 50.65 (5.61) Con: 43.98 (4.36) *ARM: MSP (mmHg)* Pre Exp: 86.32 (8.84) Con: 85.68 (4.31) Post (9 months) Exp: 110.8 (6.14) Con: 102.14 (3.48) *ARM: RRP (mmHg)* Pre Exp: 2.66 (0.89) Con: 2.61 (0.86) Post (9 months) Exp: 3.74 (1.52) Con: 3.32 (1.26)
Zheng [26], 2019, China	Exp: 54.34 (9.94) Con: 52.50 (10.44) Exp: *n* = 35 Con: *n* = 36	PFMT - Contract 5–10 s/rep × 10 reps/session × 5 sessions/day × 13 months EMG-BFT 20 min/session × 3 sessions/week, × 16 weeks	PFMT - Contract 5–10 s/rep × 10 reps/session × 5 sessions/day × 13 months	FI: ARM (ARP, MSP, RRP)	*ARM: ARP (mmHg)* Pre Exp: 44.63 (8.71) Con: 44.31 (6.69) Post (13 months) Exp: 44.83 (9.01) Con: 42.92 (7.15) *ARM: MSP (mmHg)* Pre Exp: 131.66 (11.61) Con: 131.08 (12.89) Post (13 months) Exp: 130.46 (10.00) Con: 128.36 (9.91) *ARM: RRP (mmHg)* Pre Exp: 4.71 (2.24) Con: 4.58 (2.26) Post (13 months) Exp: 4.31 (1.75) Con: 5.72 (1.85)

*ARM*, anorectal manometry; *ARP*, anal resting pressure; *BFT*, biofeedback therapy; *CCIS*, Cleveland Clinic Incontinence Score; *Con*, control group; *EMG*, electromyography; *ES*, electrical stimulation; *Exp*, experimental group; *FI*, fecal incontinence; *FIQL*, Fecal Incontinence Quality of Life scale; *IQR*, interquartile range; *MSP*, maximum squeeze pressure; *NR*, not reported; *PFMT*, pelvic floor muscle training; *Pre*, before therapy; *Post*, after therapy; *QoL*, Quality of life; *RRP*, resting rectal pressure

^*^Individual mean and standard deviation (SD) were not reported

### Risk of bias in the included trials

**Fig. 2:**
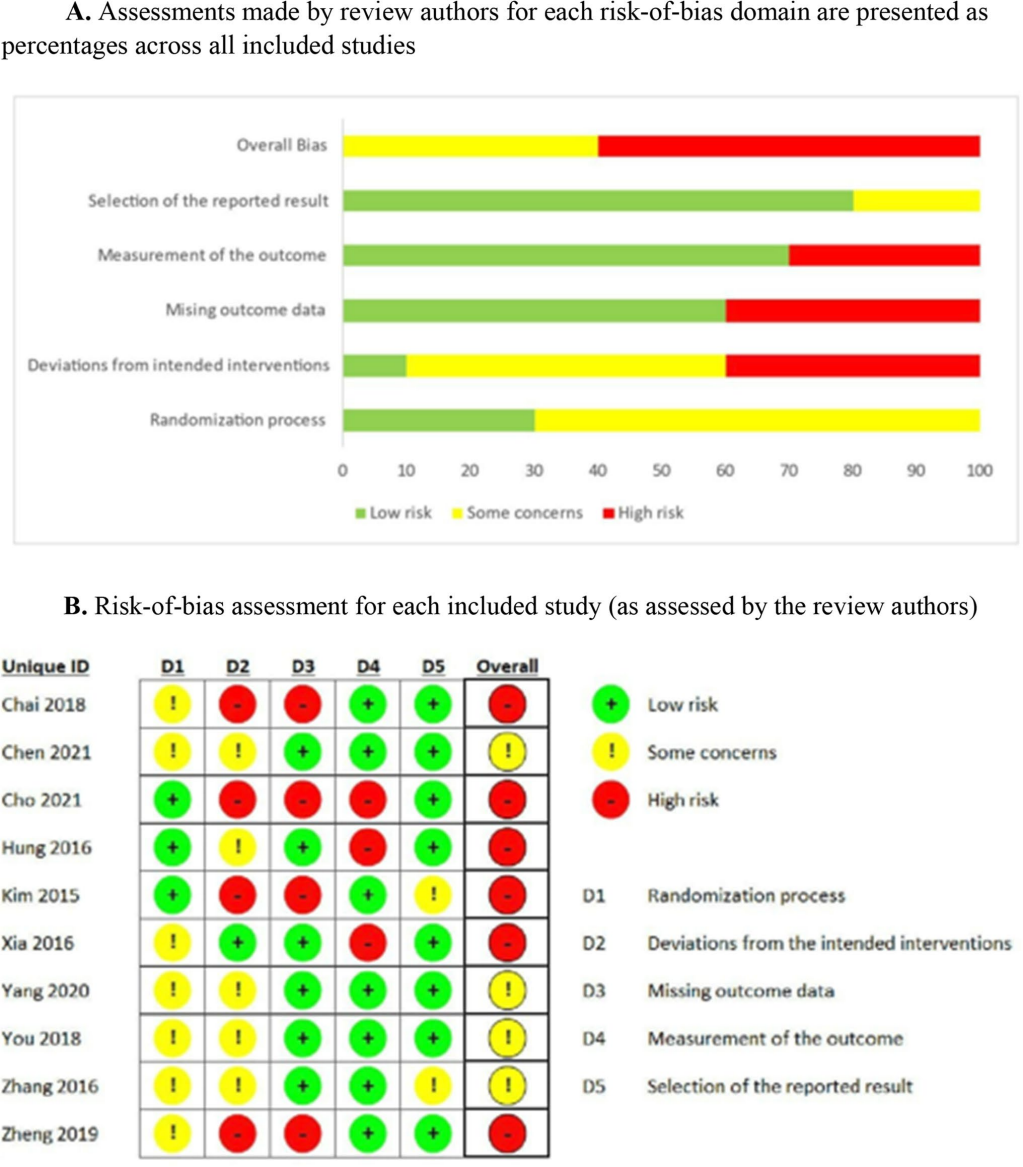
**A** Assessments made by review authors for each risk-of-bias domain are presented as percentages across all included studies. **B** Risk-of-bias assessment for each included study (as assessed by the review authors)

Figure 2A shows the distributions of RoB levels across each domain, and Fig. 2B shows the overall RoB levels assessed for all included trials. Of the 10 included trials, three (30%) reported adequate randomization processes, one (10%) had few to no deviations from the intended intervention, six (60%) were free of missing outcome data, seven (70%) blinded the outcome assessors, and eight (80%) had reported all planned outcomes. Of the 10 included trials, four had moderate RoB, and six had high RoB.

### Effects of interventions on QoL among individuals with FI after CRC surgery

#### PFMT versus UC

**Fig. 3:**
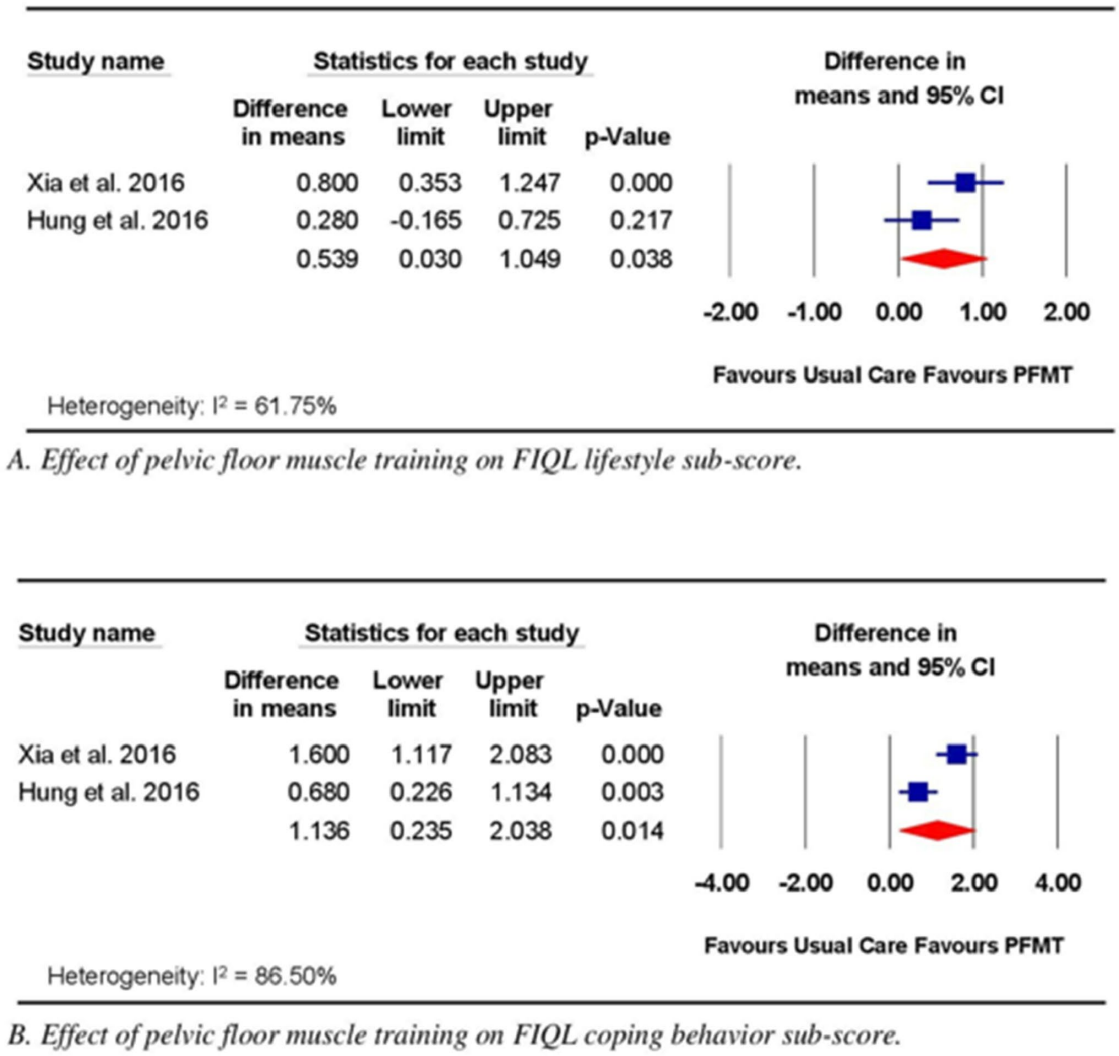

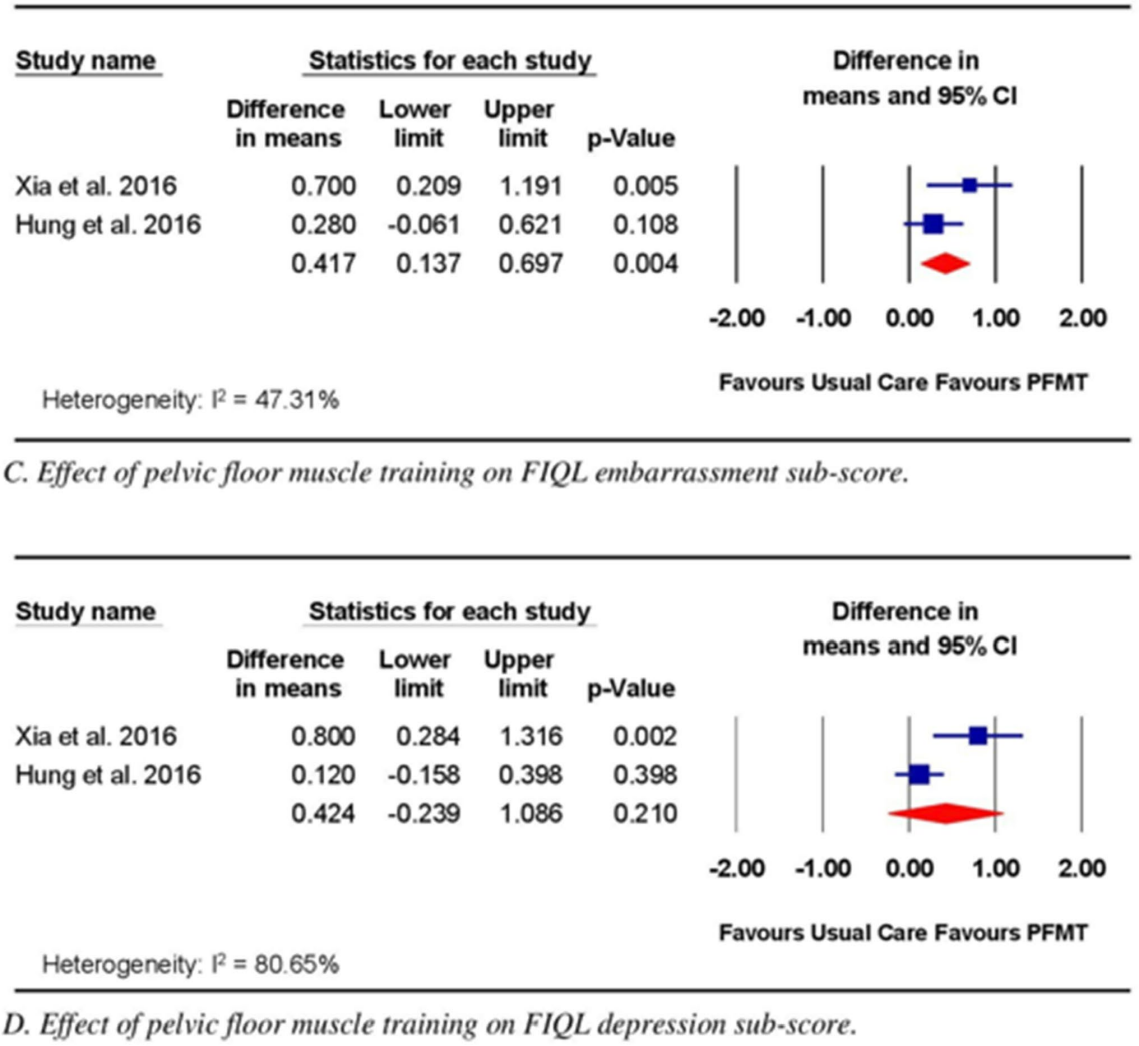
Effect of pelvic floor muscle training on quality-of-life measures assessed using Fecal Incontinence Quality of Life (FIQL) subscales

Two trials compared the effects of PFMT with those of UC on QoL [20, 21]. The RoB for these two trials was high. Both trials [20, 21] measured QoL using the FIQL and reported the lifestyle, coping behavior, depression, and embarrassment components. PFMT was performed daily in both trials, but the protocols varied. In Hung, Lin (20), participants performed four PMFT sessions per day, with each session consisting of 20 contractions. In Xia et al. [21], participants performed three PMFT sessions per day, with each session consisting of 20 contractions. The duration of PFMT ranged from 3 to 9 months. Meta-analysis of data from the two trials [20, 21] (*n* = 112) revealed significant effects of the intervention compared with UC for the lifestyle (WMD 0.54; 95% CI 0.03, 1.05; *p* = 0.04; Fig. 3A), coping behavior (WMD 1.14; 95% CI 0.24, 2.04; *p* = 0.01; Fig. 3B), and embarrassment (WMD 0.417; 95% CI 0.14, 0.70; *p* = 0.00; Fig. 3C) components of the FIQL; however, no significant effect was observed for PFMT compared with UC on the depression component (WMD 0.424; 95% CI − 0.24, 1.09; *p* = 0.21; Fig. 3D).

### Effect of interventions on FI

#### Biofeedback versus UC

**Fig. 4:**
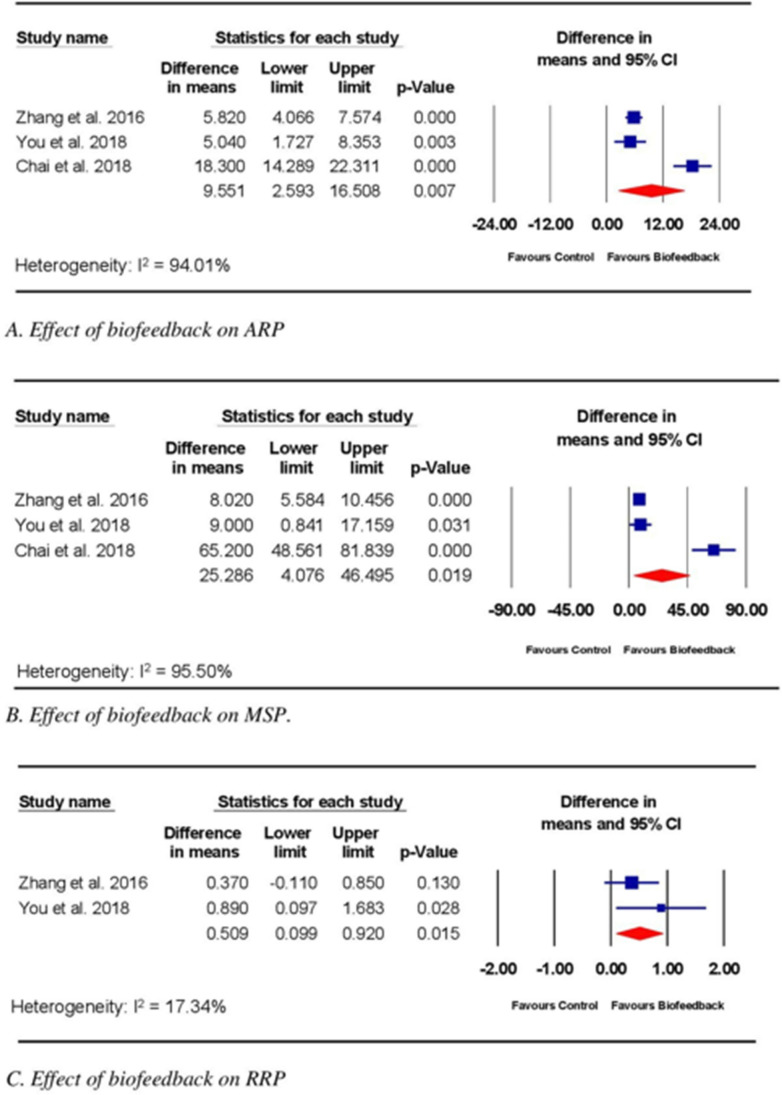
Effect of biofeedback on fecal incontinence measures assessed by anorectal manometry. Abbreviations: ARP, anal resting pressure; MSP, maximum squeeze pressure; RRP, rectal resting pressure; CI, confidence interval

Three trials compared the effects of biofeedback with those of UC on FI, measured by ARM [22–24]. The RoB for these three trials ranged from moderate to high. ARP and MSP were reported in all three trials, but only two trials reported RRP [22, 24]. In all three trials [22–24], electromyography (EMG) biofeedback therapy was implemented after colorectal surgery. One [22] of the three trials used an anal electrode inserted into the lower rectum, with adhesive electrodes placed on the external oblique muscles, forming a circuit to enable the detection of muscle activities during bowel movements. In this trial, daily biofeedback therapy, provided for 45–60 min per session, was performed for 15 days. Two trials [23, 24] provided insufficient descriptions of the method used to detect electrical activity during bowel movements or the treatment parameters. The meta-analysis of all three trials [23, 24] (*n* = 226) demonstrated a significant effect for biofeedback compared with UC for improving ARP (WMD 9.55; 95% CI 2.59, 16.51; *p* = 0.01; Fig. 4A) and MSP (WMD 25.29; 95% CI 4.08, 48.50; *p* = 0.02; Fig. 4B). The meta-analysis of the two trials reporting RRP [22, 24] (*n* = 152) found a significant effect for biofeedback compared with UC on RRP improvement (WMD 0.51; 95% CI 0.10, 0.92; *p* = 0.02; Fig. 4C).

#### PFMT plus biofeedback versus PFMT

**Fig. 5:**
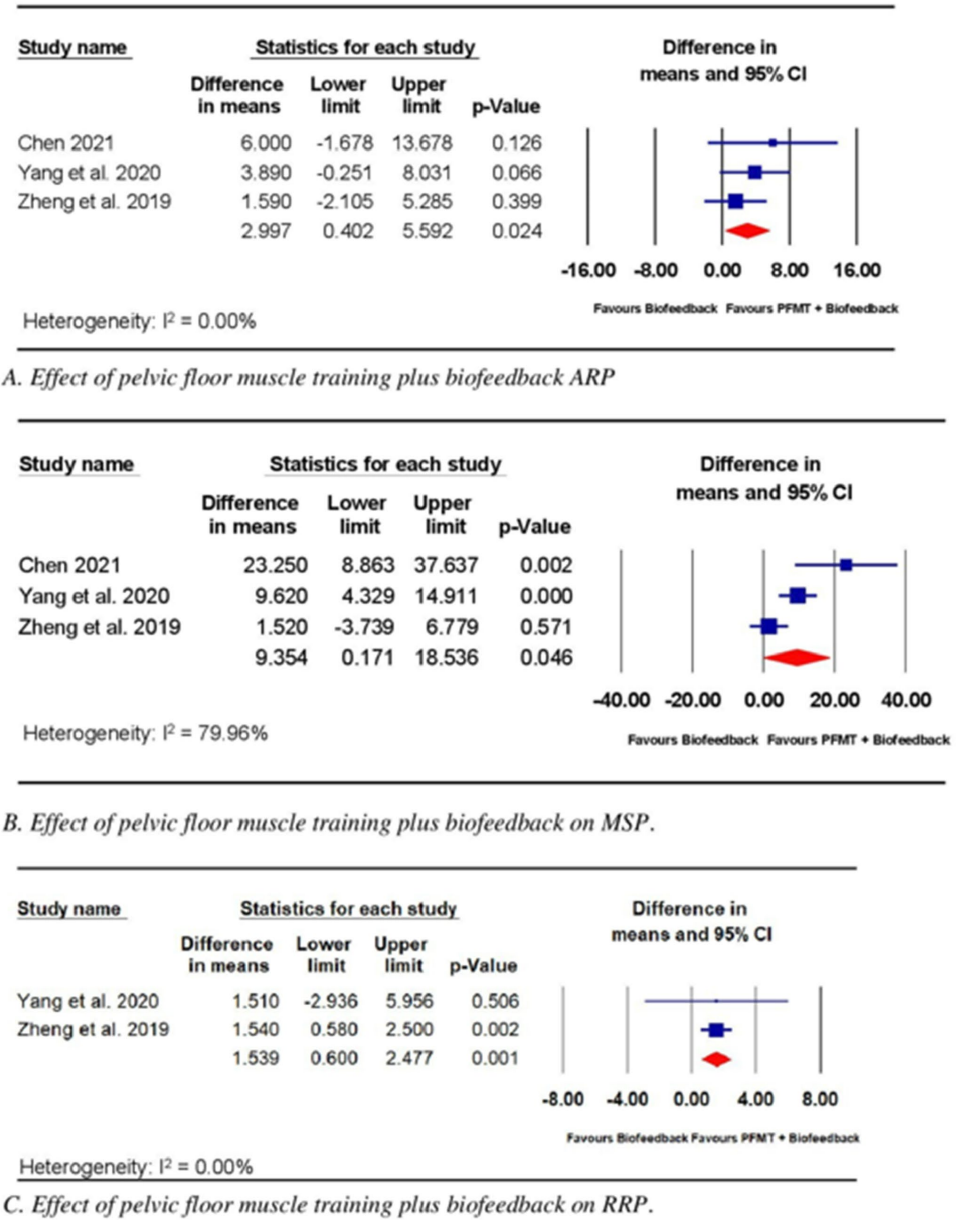
Effect of pelvic floor muscle training plus biofeedback on fecal incontinence measures assessed by anorectal manometry. Abbreviations: ARP, anal resting pressure; MSP, maximal squeeze pressure; RRP, rectal resting pressure

The effects of PFMT alone compared with the effects of PFMT combined with biofeedback on anorectal dynamics were examined in three trials [18, 25, 26]. The RoB of these three trials varied from moderate to high. All three trials [25, 26] reported ARP and MSP as outcomes, but only two trials [25, 26] reported RRP. Chen (18) and Zheng, Wu (26) included EMG biofeedback therapy in addition to PFMT. The electrical activities of the pelvic floor muscles were measured by anal electrodes in all three trials. Zheng, Wu (26) also used adhesive electrodes to detect the electrical activity of external oblique muscles. Biofeedback therapy was performed two to three times per week for 20–30 min each time, with the total training period ranging from 3 to 13 months. In the trials by Chen (18) and Zheng, Wu (26), PFMT was performed daily, consisting of five sets of 10 repetitions consisting of contractions lasting 5–10 s per repetition, with 10 s of rest between repetitions. Subjects in the study by Yang, Wang (25) performed PFMT by contracting pelvic floor muscles for 10 s. The total PMFT training period varied from 16 months to the time subjects needed to be discharged [18, 25, 26]. The meta-analysis of the three trials [18, 25, 26] (*n* = 211) showed a significant effect for biofeedback combined with PFMT compared with PMFT alone on ARP (WMD 3.00; 95% CI 0.40, 5.59; *p* = 0.02, Fig. 5A) and MSP (WMD 9.35; 95% CI 0.17, 18.53; *p* = 0.05, Fig. 5B). The meta-analysis of the two trials reporting RRP [25, 26] (*n* = 135) revealed a significant effect for biofeedback combined with PMFT compared with PMFT alone on RRP (WMD 1.54; 95% CI 0.60, 2.48; *p* = 0.00, Fig. 5C).

#### Biofeedback versus PFMT

**Fig. 6:**
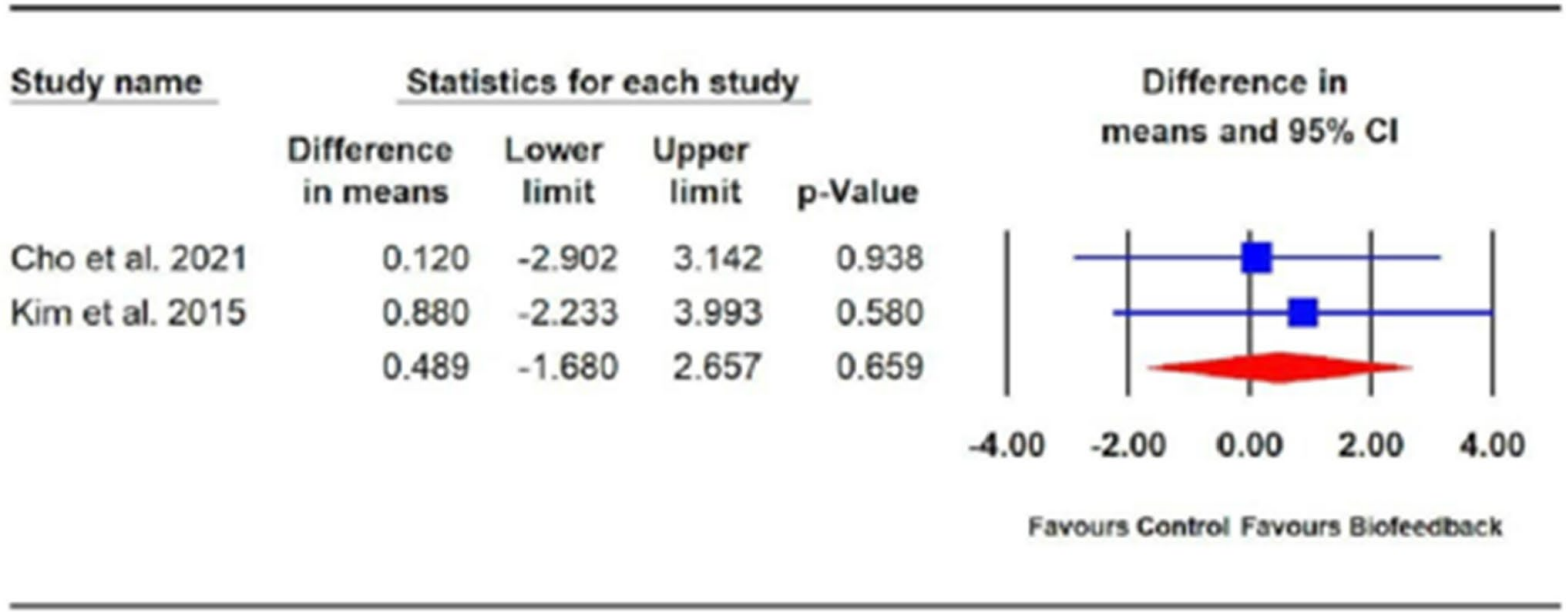
Effect of biofeedback on fecal incontinence measured by Cleveland Clinic Incontinence Score

The effects of biofeedback therapy alone were compared with the effects of PFMT alone on FI using the CCIS in two trials [27, 28]. The RoB for the two trials varied from moderate to high. Both trials provided biofeedback therapy for the experimental group, whereas the control group performed Kegel exercises. Cho, Kim (28) provided the experimental group with biofeedback training designed to strengthen their external anal sphincter. Subjects were educated to slowly contract and relax the pelvic floor muscles and were presented with visual or audible signals proportional to their anal squeezing pressure. The training was provided one to two times per week for 6 months. Kim, Jeon (27) provided the experimental group with biofeedback training in which an anorectal probe was used to train subjects to achieve adequate squeeze pressure using a visual feedback display. Each training session lasted 10 to 30 min, and subjects were encouraged to repeat the exercises five times each day. The PFMT dosage used for the control groups was not specified in either trial [27, 28]. The meta-analysis of data from both trials [27, 28] (*n* = 168) showed a non-significant effect for biofeedback alone compared with PFMT alone on the CCIS (WMD 0.49; 95% CI − 1.68, 2.66; *p* = 0.66; Fig. 6).

## Discussion

This systematic review and meta-analysis investigated the effectiveness of physiotherapy interventions on FI and QoL following colorectal surgery. The literature searches identified 4413 potentially relevant articles indexed in both English- and Chinese-language databases; however, only 10 trials met the pre-defined inclusion criteria for the meta-analysis. The interventions examined in the included trials were PFMT alone, biofeedback therapy alone, and the combination of PFMT and biofeedback therapy. The RoB of the included trials ranged from moderate to high.

Meta-analysis of data from two trials [20, 21] comparing PFMT alone with UC revealed a significant effect of the intervention on QoL components, including lifestyle, coping behavior, and embarrassment, as measured using the FIQL. The minimal clinically important difference (MCID) values reported for lifestyle, coping behavior, and embarrassment are 0.2, 0.3, and 0.2, respectively, among the noncancerous population [29]. The mean estimated effects obtained in the studies included in the current review surpassed the MCID for lifestyle (0.54), coping behavior (1.14), and embarrassment (0.43), indicating that these effects might be clinically meaningful. Despite the significant results obtained for QoL in the current review, the findings are limited by the high RoB, the limited number of pooled trials (*n* = 2), and the varying PFMT protocols used by each included study. Nevertheless, considering the effect size and safety of PFMT [30], this intervention should be considered a potential treatment option for improving QoL among individuals who experience FI following CRC surgery.

Meta-analysis of data from trials with moderate to high RoB comparing biofeedback alone with UC revealed significant effects of the intervention on the ARP, MSP [22–24], and RRP [22, 24] measures of ARM. Meta-analysis of data from trials [18, 25, 26] with moderate to high RoB identified similar significant effects on FI parameters when PFMT combined with biofeedback was compared with PFMT alone. ARM is a non-invasive procedure used to objectively quantify anorectal function and has been found to be clinically relevant for assessing the severity of FI in both children and adults [31–33]. No MCID has been established for ARM, preventing interpretation of the estimated effect size. However, the 95% CI values for both interventions (PFMT plus biofeedback and biofeedback alone) were below the MCID, indicating the potential for clinically trivial effects. Additional data examining the effects of these interventions would narrow the 95% CI and provide more precise estimates of the average effects of PFMT plus biofeedback and biofeedback alone for the treatment of FI following CRC surgery.

The results obtained in the current study for PFMT plus biofeedback agree with results reported in previous systematic reviews [34, 35] examining the effects of pelvic floor rehabilitation, including PFMT plus biofeedback, on improving anorectal function following rectal resection surgery. However, these prior systematic reviews [34, 35] did not include quantitative analyses, and both reviews included non-RCTs. By contrast, the current review quantitatively evaluated the efficacy of PFMT plus biofeedback and only included RCTs, offering a higher level of evidence [36].

Although the current review found significant effects for biofeedback alone and PFMT plus biofeedback on FI as measured by ARM, these findings are limited by a considerably high RoB, large variations in the PFMT and biofeedback protocols described in the included trials, and the small number of trials included in the meta-analysis. However, considering the non-invasive nature of PFMT and the minimally invasive nature of biofeedback, both PFMT combined with biofeedback and biofeedback alone should be considered potential interventions for improving FI following CRC surgery. Future studies should investigate additional ARM other than ARP, MSP, and RRP, such as urge volume and volume of first sensation [37], to obtain a more holistic understanding of the effects of these interventions on FI.

Meta-analysis of data from two trials [27, 28] comparing biofeedback alone with a PMFT alone revealed a non-significant effect of the intervention on FI following colorectal surgery. Based on these results, no recommendations can be made regarding the effectiveness of biofeedback alone compared with PFMT alone. Future studies of high methodological rigor are required to confirm the results obtained in this review for biofeedback alone compared with PFMT alone.

### Strengths and limitations of the review

The current review has several strengths, including being the first review to include meta-analyses evaluating the effectiveness of various physiotherapy interventions on FI and QoL following CRC surgery. A comprehensive search strategy was applied to identify RCTs evaluating the effectiveness of various physiotherapy interventions for the treatment of FI following CRC surgery. Meta-analyses revealed significant effects for PFMT alone, biofeedback alone, and the combination of PFMT with biofeedback for improving QoL and FI following CRC surgery.

Our review also has some limitations. More than half of the included studies (6 of 10) were identified as having a high RoB. Other limitations are the inclusion of unpublished theses, which might hinder the study quality because unpublished studies may be of lower methodological quality than published studies [38], heterogeneity in terms of PFMT and biofeedback protocols utilized in the included trials, which minimizes the applicability of these findings to clinical settings; a high degree of statistical heterogeneity was evident across the pooled estimates as indicated by large *I*^2^ values, small sample sizes in some of the included trial;, and the small number of trials included in meta-analyses.

## Conclusion

The current systematic review and meta-analysis identified significant improvements in the lifestyle, coping behavior, and embarrassment components of the FIQL among patients who received PFMT compared with those who received UC. Considering the non-invasive nature of PFMT and the sizes of the effects obtained for this intervention on different QoL components, PFMT should be considered an intervention that may improve QoL among individuals who experience FI following CRC surgery. Meta-analysis revealed that biofeedback alone was superior to UC and that PFMT plus biofeedback was superior to PFMT alone, with both superior interventions resulting in significant improvements in ARP, MSP, and RRP when assessed using ARM. Biofeedback is a minimally invasive intervention that can be applied alone or in combination with PFMT to treat FI following CRC surgery. However, the efficacy of biofeedback alone compared with PFMT alone remains inconclusive. Future high-quality RCTs remain necessary to standardize and optimize PFMT and biofeedback parameters for FI rehabilitation following CRC surgery and to confirm the results obtained in this review.

### Supplementary Information

Below is the link to the electronic supplementary material.

Supplementary file1 (DOCX 27 KB)
