# Supplementary material for: The effectiveness of physiotherapy interventions on fecal incontinence and quality of life following colorectal surgery: a systematic review and meta-analysis of randomized controlled trials
Source: Support Care Cancer. 2024 Jan 13;32(2):103. doi: 10.1007/s00520-023-08294-1 (PMC10787910; doi:10.1007/s00520-023-08294-1)
Supplement: Supplementary file 1 — Supplementary file1 (DOCX 27 KB) [file 520_2023_8294_MOESM1_ESM.docx]

**Supplementary Appendix 1.** Search terms and search strategy for Medline

| **Subject areas** | **Search terms adopted** | **Limits** |
| --- | --- | --- |
| Physiotherapy intervention (AND) | physiotherapy (OR) physical therapy (OR) physical therapies OR physical modality (OR) physical modalities (OR) acupuncture (OR) dry needling (OR) electroacupuncture (OR) acupressure (OR) massage (OR) manual therapy (OR) mobilization (OR) electrotherapy (OR) electrophysical agent (OR) anal stimulation (OR) biofeedback (OR) EMG biofeedback (OR) Electromyography biofeedback (OR) nerve stimulation (OR) electrical stimulation (OR) electrostimulation (OR) neuromuscular electrical stimulation (OR) transcutaneous electrical nerve stimulation (OR) extracorporeal magnetic innervation (OR) functional training (OR) exercise (OR) aerobic exercise (OR) endurance exercise (OR) cardiorespiratory (OR) cardiopulmonary exercise (OR) cardiovascular exercise (OR) stretching exercise (OR) resistance exercise (OR) strengthening exercise (OR) pelvic floor exercise (OR) pelvic floor muscle training (OR) pelvic floor rehabilitation (OR) Kegel exercise (OR) Kegel training (OR) sphincter exercise (OR) anal muscle training (OR) yoga (OR) pilates | - Human studies - Full-text RCTs - English or Chinese (both traditional and simplified) language studies published in peer-reviewed journals |
| Colorectal cancer and surgery (AND) | colorectal cancer (OR) colorectal neoplasms (OR) colorectal tumor (OR) colorectal metastasis (OR) colorectal cancroid (OR) colorectal carcinoma (OR) colorectal malignancy (OR) colorectal polyps (OR) colorectal adenoma (OR) rectal cancer (OR) rectal neoplasms (OR) rectal tumor (OR) rectal metastasis (OR) rectal cancroid (OR) rectal carcinoma (OR) rectal malignancy (OR) rectal polyps (OR) rectal adenoma (OR) bowel cancer (OR) bowel neoplasms (OR) bowel tumor (OR) bowel metastasis (OR) bowel cancroid (OR) bowel carcinoma (OR) bowel malignancy (OR) bowel polyps (OR) bowel adenoma (OR) anal cancer (OR) anal neoplasms (OR) anal neoplasia (OR) anal tumor (OR) anal canal tumor (OR) anal carcinoma (OR) anus cancer (OR) anus neoplasms (OR) anus tumor (OR) anus metastasis (OR) anus cancroid (OR) anus carcinoma (OR) anus malignancy (OR) anus polyps (OR) anus adenoma (OR) anorectal cancer (OR) anorectal neoplasms (OR) anorectal tumor (OR) anorectal metastasis (OR) anorectal cancroid (OR) anorectal carcinoma (OR) anorectal malignancy (OR) anorectal polyps (OR) anorectal adenoma (OR) serrated adenoma (OR) Peutz Jeghers syndrome (OR) hyperplastic polyp (OR) juvenile polyp (OR) juvenile polyposis syndrome (OR) familial adenomatous polyposis (OR) Gardner syndrome (OR) polymerase proofreading associated polyposis (OR) serrated polyposis (OR) squamous cell carcinoma (OR) colorectal cancer surgery (OR) total mesorectal excision (OR) sphincter preserving surgery (OR) sphincter saving surgery (OR) anorectal surgery (OR) anterior resection (OR) low anterior resection (OR) abdominoperineal resection (OR) colorectal resection (OR) colectomy (OR) hemicolectomy (OR) partial colectomy (OR) segmental resection (OR) colorectal surgery (OR) laparoscopy (OR) laparoscopic (OR) colonoscopy (OR) transanal endoscopic microsurgery (OR) sigmoidoscopy (OR) Piecemeal mucosectomy (OR) submucosal dissection (OR) endoscopic submucosal dissection (OR) transanal microsurgery (OR) transanal endoscopic microsurgery (OR) polypectomy (OR) protocolectomy (OR) radiation (OR) radiotherapy (OR) radiation therapy (OR) radioembolization O (OR) R chemoradiotherapy (OR) External beam radiation therapy (OR) three dimensional conformal radiation therapy (OR) intensity modulated radiation therapy (OR) Image guided radiation therapy (OR) stereotactic body radiation therapy (OR) Internal radiation therapy (OR) Endocavitary radiation therapy (OR) Intraoperative radiation therapy (OR) brachytherapy (OR) neoadjuvant therapy |  |
| Bowel incontinence (AND) | anal incontinence (OR) accidental bowel leakage (OR) bowel incontinence (OR) bowel control (OR) defecation (OR) encopresis (OR) feces (OR) functional encopresis (OR) fecal incontinence (OR) feces incontinence (OR) incontinence (OR) Gatism (OR) soiling |  |
| Randomized controlled trial | randomized control trial (OR) randomized controlled trial (OR) randomized controlled clinical trial (OR) RCT (OR) randomization (OR) clinical trial (OR) random allocation |  |

**Supplementary Appendix 2.** Excluded studies and reasons for exclusion in full-text screening

| **No.** | **First author and year** | **Study title** | **Reasons for exclusion** |
| --- | --- | --- | --- |
| 1 | Mai, 2017 | [Influence of electroacupuncture pretreatment on intestinal function in the patients of colorectal cancer surgery] | Pre-treatment |
| 2 | López-Rodríguez-Arias, 2021 | Effect of home-based prehabilitation in an enhanced recovery after surgery program for patients undergoing colorectal cancer surgery during the COVID-19 pandemic | Pre-treatment |
| 3 | Bartlett, 2011 | Biofeedback for fecal incontinence: a randomized study comparing exercise regimens | Ineligible participants* |
| 4 | Bartlett, 2011 | Biofeedback therapy for faecal incontinence: a rural and regional perspective | Ineligible participants* |
| 5 | Bartlett, 2012 | Impact of relaxation breathing on the internal anal sphincter in patients with faecal incontinence | Ineligible participants* |
| 6 | Bartlett, 2015 | Supplementary Home Biofeedback Improves Quality of Life in Younger Patients With Fecal Incontinence | Ineligible participants* |
| 7 | Bols, 2012 | Rectal balloon training as add-on therapy to pelvic floor muscle training in adults with fecal incontinence: a randomized controlled trial | Ineligible participants* |
| 8 | Byrne, 2005 | Telephone vs. face-to-face biofeedback for fecal incontinence: comparison of two techniques in 239 patients | Ineligible participants* |
| 9 | Davis, 2004 | Adjuvant biofeedback following anal sphincter repair: a randomized study | Ineligible participants* |
| 10 | Efverman, 2020 | Treatment expectations seem to affect bowel health when using acupuncture during radiotherapy for cancer: Secondary outcomes from a clinical randomized sham-controlled trial | Ineligible participants* |
| 11 | Healy, 2006 | The effects of low-frequency endo-anal electrical stimulation on faecal incontinence: a prospective study | Ineligible participants* |
| 12 | Laurienzo, 2013 | Results of preoperative electrical stimulation of pelvic floor muscles in the continence status following radical retropubic prostatectomy | Ineligible participants* |
| 13 | Liu, 2021 | Electroacupuncture vs Prucalopride for Severe Chronic Constipation: A Multicenter, Randomized, Controlled, Noninferiority Trial | Ineligible participants* |
| 14 | Meng, 2010 | Electro-acupuncture to prevent prolonged postoperative ileus: a randomized clinical trial | Ineligible participants* |
| 15 | Özdemir, 2019 | Impact of pre-operative walking on post-operative bowel function in patients with gynecologic cancer | Ineligible participants* |
| 16 | Rao, 2021 | Translumbosacral Neuromodulation Therapy for Fecal Incontinence: A Randomized Frequency Response Trial | Ineligible participants* |
| 17 | Yang, 2022 | Effect of acupuncture on postoperative ileus after laparoscopic elective colorectal surgery: A prospective, randomised, controlled trial | Ineligible participants* |
| 18 | Andy, 2018 | Impact of treatment for fecal incontinence on defecatory symptoms | No full-text |
| 19 | Cho, 2016 | The effect of biofeedback therapy during interval of temporary stoma on anorectal function: the interim report of randomized controlled study | No full-text |
| 20 | Duchalais, 2019 | Impact of pelvic floor prehabilitation using biofeedback therapy on the severity of low anterior resection syndrome following total mesorectal excision: CONTICARE randomized controlled trial | No full-text |
| 21 | Ax, 2022 | Short-and long-term effect of high versus low-to-moderate intensity exercise to optimise health-related quality of life after oncological treatment—results from the Phys-Can project | Ineligible outcome measures# |
| 22 | Blank, 2019 | Impact of auricular neurostimulation in patients undergoing colorectal surgery with an enhanced recovery protocol: a randomized, controlled trial | Ineligible outcome measures# |
| 23 | Chao, 2013 | The Beneficial Effect of ST-36 (Zusanli) Acupressure on Postoperative Gastrointestinal Function in Patients With Colorectal Cancer | Ineligible outcome measures# |
| 24 | Cheng, 2017 | Effects of Kegel on fecal incontinence in patients with anal endoscopic microsurgery after surgery | Ineligible outcome measures# |
| 25 | Deng, 2013 | A Phase II, Randomized, Controlled Trial of Acupuncture for Reduction of Postcolectomy Ileus | Ineligible outcome measures# |
| 26 | Liu, 2019 | The effect of biofeedback training on intestinal function among patients with middle and low rectal cancer: a randomized controlled study | Ineligible outcome measures# |
| 27 | Kai, 2017 | Perioperative Transcutaneous Electrical Acupoint Stimulation for Postoperative Pain Relief Following Laparoscopic Surgery: A Randomized Controlled Trial | Ineligible outcome measures# |
| 28 | Mathias, 2022 | Effect of TENS and stabilization exercises on pelvic pain in pelvic cancer survivors following multimodal treatment: A clinical trial | Ineligible outcome measures# |
| 29 | Ozhanli, 2022 | The effect of progressive relaxation exercise on physiological parameters, pain and anxiety levels of patients undergoing colorectal cancer surgery: a randomized controlled study | Ineligible outcome measures# |
| 30 | Xiao, 2017 | The study of biofeedback and acupuncture therapy in the treatment of postoperative defecation disorders of patients with rectal cancer sphincter-sparing surgery | Ineligible outcome measures# |
| 31 | Dulskas, 2022 | The Role of Traditional Acupuncture in Low Anterior Resection Syndrome Treatment: A Pilot Study | Non-RCT |
| 32 | Harji, 2021 | A novel bowel rehabilitation programme after total mesorectal excision for rectal cancer: the BOREAL pilot study | Non-RCT |
| 33 | Ho, 1997 | Biofeedback therapy for bowel dysfunction following low anterior resection | Non-RCT |
| 34 | Kim, 2011 | Effectiveness of Biofeedback Therapy in the Treatment of Anterior Resection Syndrome After Rectal Cancer Surgery | Non-RCT |
| 35 | Kuo, 2015 | Improvement of fecal incontinence and quality of life by electrical stimulation and biofeedback for patients with low rectal cancer after intersphincteric resection | Non-RCT |
| 36 | Laforest, 2012 | Functional disorders after rectal cancer resection: does a rehabilitation programme improve anal continence and quality of life? | Non-RCT |
| 37 | Pucciani, 2008 | Rehabilitation of fecal incontinence after sphincter-saving surgery for rectal cancer: Encouraging results | Non-RCT |
| 38 | Xue, 2022 | Clinical Study of Postoperative Low Anterior Resection Syndrome of Rectal Cancer Treated by Thumb-tack Needle | Non-RCT |
| 39 | Blank, 2021 | Impact of Auricular Neurostimulation in Patients Undergoing Colorectal Surgery with an Enhanced Recovery Protocol: a Pilot Randomized, Controlled Trial | Study protocol |
| 40 | Kalkdijk-Dijkstra, 2020 | Pelvic floor rehabilitation to improve functional outcome and quality of life after surgery for rectal cancer: study protocol for a randomized controlled trial (FORCE trial) | Study protocol |
| 41 | Sacomori, 2021 | A randomized clinical trial to assess the effectiveness of pre- and post-surgical pelvic floor physiotherapy for bowel symptoms, pelvic floor function, and quality of life of patients with rectal cancer: CARRET protocol | Study protocol |
| 42 | Asnong, 2022 | Asnong A, D’Hoore A, van Kampen M, Wolthuis A, van Molhem Y, van Geluwe B, et al. The role of pelvic floor muscle training on low anterior resection syndrome a multicenter randomized controlled trial | Unable to pool |
| 43 | Brown, 2018 | A randomized dose-response trial of aerobic exercise and health-related quality of life in colon cancer survivors | Unable to pool |
| 44 | Chen, 2019 | Efficacy of biological feed back therapy in improving anal function of patients with low rectal carcinoma after anus-preserving surgery | Unable to pool |
| 45 | Gao, 2022 | Effect of biofeedback combined with electrical stimulation on anus functionin patients with anal incontinence after anus preservation for rectal cancer | Unable to pool |
| 46 | Ji, 2021 | Effects of different water volume balloon combined with pelvic floor muscle function exercise on defecation and stoma reduction after enterostomy | Unable to pool |
| 47 | Li, 2021 | Anal Function Evaluation after Anus-saving Procedure for Rectal Cancer, and Clinical Study on Pelvic Floor Muscles Exercise Promoting Anal Function Rehabilitation | Unable to pool |
| 48 | Li, 2022 | Application Experience and Effect of Pelvic Floor Function Rehabilitation Nursing in Postoperative Patients with Rectal Cancer | Unable to pool |
| 49 | Liu, 2013 | The research of protect anal rectal cancer postoperative anal function evaluation and comprehensive treatment to promote the anal function recovery | Unable to pool |
| 50 | Lin, 2016 | Effects of pelvic floor muscle exercise on faecal incontinence in rectal cancer patients after stoma closure | Unable to pool |
| 51 | Ma, 2021 | Effect of Pelvic Floor Exercise on Anal Sphincter After Operation of Intestinal Tumour | Unable to pool |
| 52 | Morielli, 2021 | Effects of exercise during and after neoadjuvant chemoradiation on symptom burden and quality of life in rectal cancer patients: a phase II randomized controlled trial | Unable to pool |
| 53 | Pilkington, 2011 | Biofeedback for improving anal continence after major rectal resection for cancer | Unable to pool |
| 54 | Ren, 2022 | Clinical study on acupuncture Baliao points combined with biofeedback in the treatment of low anterior resection syndrome after operation for rectal cancer | Unable to pool |
| 55 | Wang, 2020 | Effects of biomechatronic feedback combined with pelvic floor muscle exercise on postoperative ano rectum function indexes in patients with low-middle rectal cancer | Unable to pool |
| 56 | Xu, 2013 | The development owning to acupuncture in defecation function of patients after undergoing anterior resection for rectal cancer: a clinical study | Unable to pool |
| 57 | Yang, 2018 | Effect of pelvic floor muscle training for fecal incontinence in patients undergoing enterostomy | Unable to pool |
| 58 | Ma, 2022 | The significance of pelvic floor muscle function training on fecal incontinence in patients with middle and low rectal cancer after sphincter preservation | Insufficient data |
| 59 | van der Heijden, 2021 | Pelvic Floor Rehabilitation After Rectal Cancer Surgery: A Multicentre Randomised Clinical Trial (FORCE Trial) | Insufficient data |
| 60 | Zhang, 2022 | Evaluation of Nursing Effects of Pelvic Floor Muscle Rehabilitation Exercise on Gastrointestinal Tract Rectal Cancer Patients Receiving Anus-preserving Operation by Intelligent Algorithm-based Magnetic Resonance Imaging | Insufficient data |
| 61 | Chan, 2022 | Chan H, Van Loon K, Kenfield SA, Chan JM, Mitchell E, Zhang L, et al. Quality of life of colorectal cancer survivors participating in a pilot randomized controlled trial of physical activity trackers and daily text messages | Insufficient data |

Note: *Ineligible participants: without colorectal cancer; #Ineligible outcome measures: outcome measures outside of the scope of interest of the current meta-analytic review.
